# Emerging Options for the Diagnosis of Bacterial Infections and the Characterization of Antimicrobial Resistance

**DOI:** 10.3390/ijms22010456

**Published:** 2021-01-05

**Authors:** Simone Rentschler, Lars Kaiser, Hans-Peter Deigner

**Affiliations:** 1 Institute of Precision Medicine, Furtwangen University, Jakob-Kienzle-Straße 17, 78054 VS-Schwenningen, Germany; s.rentschler@hs-furtwangen.de (S.R.); la.kaiser@hs-furtwangen.de (L.K.); 2 Department of Pharmaceutical and Medicinal Chemistry, Institute of Pharmaceutical Sciences, Eberhard Karls University Tübingen, Auf der Morgenstelle 8, 72076 Tübingen, Germany; 3 Institute of Pharmaceutical Sciences, University of Freiburg, Albertstraße 25, 79104 Freiburg i. Br., Germany; 4 EXIM Department, Fraunhofer Institute IZI (Leipzig), Schillingallee 68, 18057 Rostock, Germany; 5 Faculty of Science, Tuebingen University, Auf der Morgenstelle 8, 72076 Tübingen, Germany

**Keywords:** infectious disease, bacterial infections, pathogen identification, resistance profiling, antimicrobial susceptibility testing, point-of-care diagnosis, precision medicine

## Abstract

Precise and rapid identification and characterization of pathogens and antimicrobial resistance patterns are critical for the adequate treatment of infections, which represent an increasing problem in intensive care medicine. The current situation remains far from satisfactory in terms of turnaround times and overall efficacy. Application of an ineffective antimicrobial agent or the unnecessary use of broad-spectrum antibiotics worsens the patient prognosis and further accelerates the generation of resistant mutants. Here, we provide an overview that includes an evaluation and comparison of existing tools used to diagnose bacterial infections, together with a consideration of the underlying molecular principles and technologies. Special emphasis is placed on emerging developments that may lead to significant improvements in point of care detection and diagnosis of multi-resistant pathogens, and new directions that may be used to guide antibiotic therapy.

## 1. Introduction

Infectious disease epidemics, emerging new diseases, as well as new and increasing problems with current methods used to treat infectious diseases, represent major challenges to our health care system. Infectious diseases remain among the leading causes of morbidity and mortality worldwide, especially in lower-income countries. Rapid, inexpensive, and readily available diagnostic techniques are required to combat this problem [1]. As infectious micro-organisms emerge over time and have the capacity to evolve, it is particularly important to develop diagnostics at a rate that will evolve rapidly to meet this challenge [2]. New clinical diagnostic methods will be needed to identify both familiar and new pathogens as part of standard clinical care; we also need methods that will determine appropriate therapy and that can evaluate the responses to therapy as well as patient prognosis [3]. Moreover, the application of the appropriate diagnostic tests will be crucial to avoid misdiagnosis and to avoid the application of therapy that is ineffective against a given pathogen. Furthermore, the inappropriate use of antibiotics contributes to the development of antimicrobial-resistant organisms [4,5,6,7].

While the use of modern molecular and biochemical technologies has resulted in the improved performance of many diagnostic modalities, diagnosis of many infectious diseases, including sepsis, pneumonia, and culture-negative endocarditis, remains difficult [8]. New tests that can be used at point-of-care (POC) are needed to improve the diagnosis of infectious diseases especially in developing countries that lack high-quality infrastructure and well-trained personnel. POC tests are designed to be used directly on-site and to provide rapid results to facilitate immediate clinical decision-making and improved clinical outcomes [9,10]. The ideal POC test would fulfill the ASSURED criteria (i.e., affordable, sensitive, specific, user-friendly, rapid and robust, equipment-free, and deliverable to end-users) recommended by the World Health Organization (WHO). Thereby, the tests can be readily applied all over the world without the need of sophisticated laboratory equipment and can easily be interpreted also by operators without in-depth knowledge on the test principle [11,12].

This article highlights the current technologies used to identify bacterial infections and to determine patterns of antimicrobial resistance (see Table 1 for an comparative overview). Findings are presented that emphasize the need for modern diagnostic options that can be used to identify emerging bacterial infections.

## 2. Methods for Identifying Infectious Agents

### 2.1. Traditional Microbiological Methods

**Figure 1:**
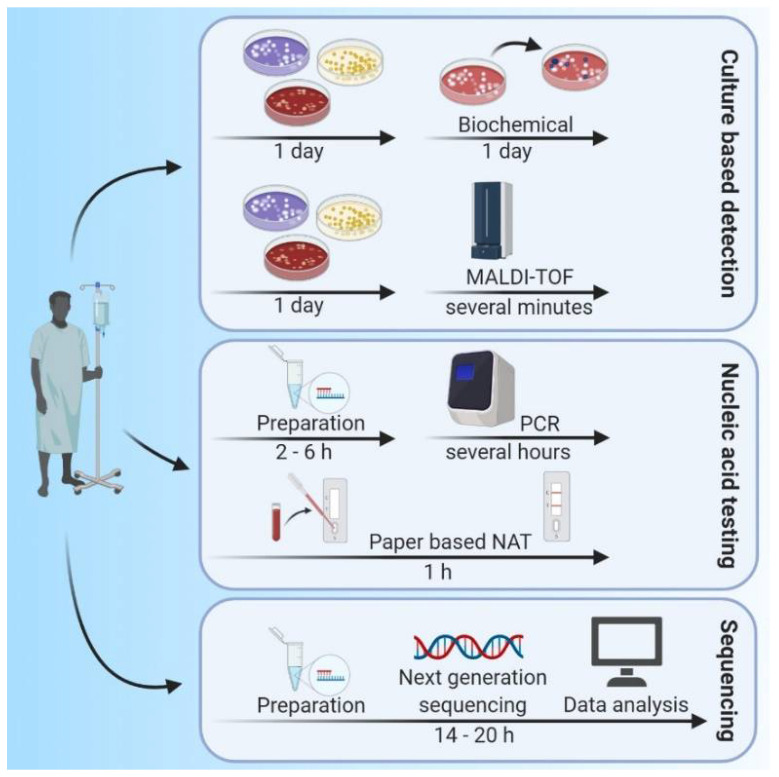
Typical timeframes required for techniques in current use for the diagnosis of bacterial infections. The classical cultivation of biological specimens, in combination with biochemical characterization, requires ~42 h as a minimum estimate. Replacement of biochemical methods with MALDI-TOF mass spectrometry (MS) reduces this timeframe significantly. The use of nucleic acid testing (NAT) bypasses the initial cultivation of clinical specimens, and thus reduces the timeframes to fewer than 4 h. However, NAT methods are sequence-dependent and involve only a limited number of primer-combinations; as such, these methods require a priori knowledge of the suspected pathogen(s). The use of NGS-based methods eliminates the need for any a priori knowledge of a suspected pathogen, although typical timeframes are increased.

For more than a century, the gold standard in bacterial infection detection relied on the growth of pathogens in cell culture, followed by evaluation using biochemical methods designed to identify strains and species of micro-organisms [13]. Bacterial cultivation is cost-effective and usually results in a diagnosis with good specificity [14]. However, this process has long turnaround times (typically 24–72 h for cultivation and an additional 18–24 h for biochemical characterization of the isolates [5]). However, these methods often lack sensitivity and generate errors associated with collection conditions and specific growth media requirements; this is particularly limiting when one needs to consider infection with one or more fastidious micro-organisms [6,13,14]. The use of modern mass spectrometry methods, including matrix-assisted laser desorption ionization time of flight (MALDI-TOF), reduces the time needed for identification of a given bacterial species from hours to several minutes (see Figure 1, Culture based detection) [15]. However, the use of MALDI-TOF still requires bacterial cultivation as a first step. One of the major drawbacks of routine bacterial culture is that these methods do not permit the identification of non-cultivable pathogen species. As such, other diagnostic methods are needed [16].

### 2.2. Molecular Methods, Polymerase Chain Reaction (PCR) and DNA Sequencing

The development of culture-independent molecular technologies, most notably polymerase chain reaction (PCR)-based diagnostics, has revolutionized the diagnosis of infectious diseases. The use of these methods can drastically reduce the time needed to obtain a diagnosis from two days or more (sometimes more than one week) to several hours (see Figure 1, Nucleic acid testing) [8,16]. The development of real-time polymerase chain reaction (RT-PCR) assays and other nucleic acid amplification methods, notably isothermal techniques (e.g., loop-mediated isothermal amplification (LAMP)), nucleic acid sequence-based amplification (NASBA), transcription-mediated amplification (TMA), and strand displacement facilitated the identification of infectious agents in clinical specimens without the need for prior cultivation [17]. Highly multiplexed PCR panels have been designed that permit simultaneous detection of the most common bacterial agents causing specific clinical syndromes [18]. Although these nucleic acid testing (NAT) methods typically perform well in the clinical setting, they are expensive, time-consuming, and require sophisticated equipment and skilled laboratory personnel. As such NAT methods are used nearly exclusively in centralized laboratories and not routinely suitable for application at POC [17,19]. Still, compared to traditional serology-based detection methods, NAT methods may provide a earlier diagnosis, due to higher sensitivity [20].

Amplification-based technologies (e.g., PCR) require a priori knowledge of suspected pathogens and their nucleic acid sequences. As many infections present with similar clinical symptoms, one or more causative micro-organisms might not be considered in the design of a specific amplification-based diagnostic test; as such, not all relevant pathogens may be identified with these methods [16,21]. This problem may be avoided by the use of non-targeted methods for pathogen identification. Next-generation sequencing (NGS) technologies have already been widely applied in micro-biology research and are now being used with increasing frequency in routine clinical micro-biological diagnostics and for monitoring of infectious diseases [22,23]. The use of metagenomic NGS (mNGS) facilitates the simultaneous and independent sequencing of billions of nucleic acid fragments contained in heterogeneous samples. Organisms can be identified without a priori knowledge at the subspecies or strain level defined by single nucleotide polymorphisms (SNPs) and other genotype variants, by aligning sequences identified to enlarging reference databases [23,24]. NGS technology, combined with bioinformatics, has become a powerful tool for rapid detection, identification, and analysis of human pathogens (see Figure 1, sequencing). These methods not only promote more accurate detection and characterization of pathogens, the use of mNGS facilitates the identification and detection of virulence and pathogenicity factors and can monitor the emergence of vaccine escape variants [25,26]. Moreover, as mNGS provides an analysis of the entire nucleic acid pool within a single biological sample, it can also provide an accurate assessment of the composition of microbial communities. These communities include many organisms that are difficult, if not impossible to identify by culture-based methods. As such, mNGS is currently the best analytical tool available for broad, comparatively unbiased identification of pathogens as well as for profiling whole bacterial micro-biomes to facilitate identification of factors associated with general health as well as various diseases [24,25].

The routine use of NGS technology by clinical micro-biology laboratories awaits the resolution of many challenges, including simplification of complex workflows and direct methods to address experimental pitfalls and biases that may be introduced in current NGS protocols (e.g., variations in sample handling and DNA extraction methods). Yet to be addressed are uniform methods to be used to resolve dominant error types (e.g., indel, substitution, and deletion) and the associated overall error rate [22,27,28]. Other concerns include ubiquitous contamination from microbial DNA found in reagents, on instruments, and introduced from the environment. Likewise, identification of pathogen signal within what is typically a vast amount of host microbial DNA present in primary specimens represents a serious hurdle for the rapid analysis of sequencing results [29]. Except for cerebrospinal fluid (CSF) and brain biopsies, all other clinically-relevant biological specimens (e.g., blood, urine) contain host-derived microbes [30], which also undergo sequencing by mNGS. Accurate evaluation of these specimens requires a means to distinguish between bacteria that normally colonize a healthy host from those associated with acute or chronic infection [31]. The translation of bacterial sequence data into medically-actionable information in a clinically relevant time frame is also among the major issues of concern [5]. The analysis of mNGS sequence data poses high demands on bioinformatics and requires substantial software support, as outcomes are generated via the use of an extensive array of algorithms that perform quality filtering, operational taxonomic unit (OTU) clustering, and sequence classification. Given the requirements for sophisticated bioinformatics support, there has only been limited application of NGS technologies outside of specialized micro-biology laboratories [22,32]. Taken together, these issues and their associated relatively high costs have impeded the use of NGS technology in routine clinical micro-biology diagnostics laboratories and certainly precludes its use in POC tests [27,31,33]. One approach that has been taken to address this issue is the MinION, a portable real-time device for DNA and RNA sequencing developed by Oxford Nanopore Technologies [34]. MinION is a nanopore-based sequencing platform that shows great potential for precise real-time analysis of both the composition and structure of complex microbial communities and pathogen identification. However, further improvements to this technology, particularly those that address sequence throughput and error-rate are necessary prior to its deployment as a clinical tool [35,36,37].

### 2.3. Mass Spectrometry (MS)

**Figure 2:**
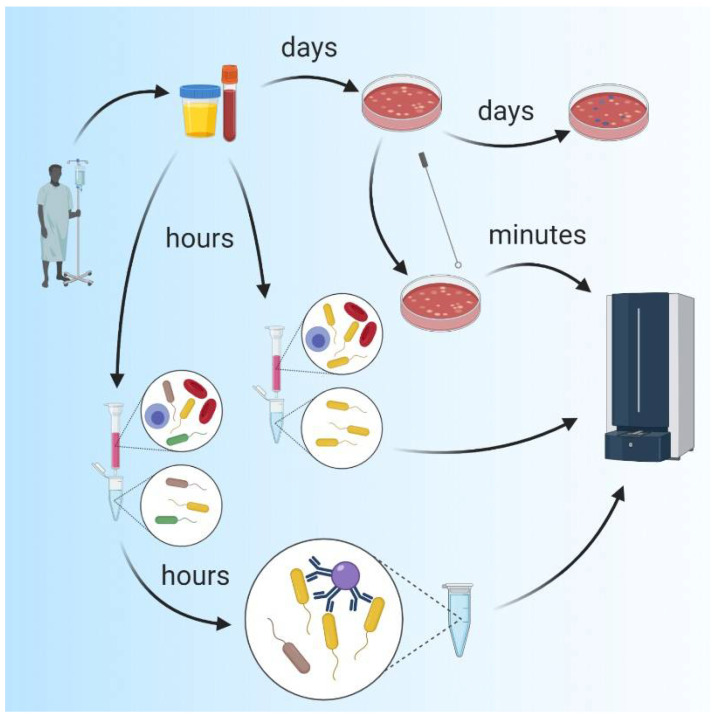
Possible shortcuts for bacterial pathogen identification from patient specimens, using MALDI-TOF MS. Typically, specimens are cultivated and identified via biochemical approaches. By MALDI-TOF-based analysis of single colonies obtained during the first round of cultivation, long turnaround times obligatory for biochemical characterization, can be bypassed. Furthermore, patient specimens can be directly analyzed by purification and concentration of bacterial cells, further shortening turnaround time. However, in case of polymicrobial infections, single bacteria need to be further separated, increasing the time to result. As polymicrobial infections cannot be ruled out in most clinical cases, a further separation of single species should be integrated into routine workflows.

The replacement of traditional biochemical diagnostic methods with MALDI-TOF MS, reduced the time required for bacterial identification from several hours to several minutes (see Figure 2) [15]. As MALDI-TOF MS is performed on an automatable, inexpensive, and rapid platform, several devices have been developed for routine use in clinical micro-biology laboratories. At the current time, the VITEK^®^ MS (bioMérieux, Inc., Durham, NC, USA) and the MALDI Biotyper CA System (Bruker Daltonics, Inc., Manning Park, MA, USA), are both approved by the US Food and Drug Administration (FDA) for identification of cultured bacteria [15,38]. During a typical analysis, bacteria are identified by their unique mass spectrum via the comparison of findings generated on-site to a database that includes spectra obtained from pure bacterial colonies. Unfortunately, this approach inevitably leads to critical restrictions in the interpretation of the data and identification of the bacterial species present in the clinical sample. First, as the database consists of spectra from pure colonies, pathogen identification typically requires a time-consuming culture step to facilitate isolation of bacterial colonies from the clinical sample. As a result, the total time-to-result is typically reduced to a comparatively limited extent, to <50 h, compared to classical biochemical methods which typically require 2–4 days to complete [38]. Furthermore, in most cases, the characterization of polymicrobial infections can only be achieved by analysis of several colonies that were selected by visual inspection of the culture plate. As this strategy often requires initial cultivation of the clinical sample, diagnosis using MALDI-TOF MS analysis will be limited to cultivable pathogens. Finally, given the minimal sample pretreatment performed in most clinical laboratories, only the most abundant molecules will be detected and identified in the spectral data, which limits the capacity to identify certain bacterial subspecies. Nonetheless, compared to methods based on phenotypical and biochemical testing, advantages of MALDI-TOF-based methods include the reduced time required for pathogen identification as well as the reduction in overall costs by 5- to 55-fold [15]. Similar results are obtained when the MALDI-TOF approach is compared to NGS-based methods.

Indeed, considerable effort has been made over the past few decades to facilitate direct testing of clinical samples by MALDI-TOF. Common approaches include affinity- or filtration-based techniques for specimen purification and cell enrichment [15,39]. For example, lectin-modified substrates can be used to select and thus enrich samples with bacterial cells, thereby facilitating their subsequent identification by MALDI-TOF [40]. Other successful enrichment strategies involved antibody—or vancomycin-coated magnetic nanoparticles which also facilitated direct pathogen identification from biological materials [41,42]. Simple and rapid diafiltration methods were successfully applied to pretreat urine specimens, thereby reducing the total time to identification to 2–3 h, which compared favorably to the 24 h to 48 h typically required for conventional culture [43]. Similar workflows should also be feasible for other less complex applications, including cerebrospinal fluid (CSF) specimens. However, direct sub-culturing or further processing may still be necessary for diagnostic analysis of complex specimens (e.g., blood or blood cultures) [15]. Readily available commercial solutions (e.g., Sepsityper from Bruker Daltonics, Inc.) can be used for this application to facilitate the use of high-throughput approaches.

It is important to recognize that several of the aforementioned techniques involve nonselective enrichment of all bacterial cells present in a biological specimen. At the current time, MALDI-TOF-based approaches are not highly effective for assessing polymicrobial samples; positive identification of a single bacterium can be achieved in 64% of the samples and several bacterial species remain unidentified [15,38,44,45,46]. Typical factors leading to false or impossible identification include the use of inappropriate software, lack of unique characters that permit differentiation between spectral signatures, and low abundance of suitable target proteins, which are then masked by high background levels [44]. To avoid this circumstance, isolate-based mixture assessment (IBMA), involving the separation of the poly-bacterial mixture into single cells and identifying each isolate separately via MALDI-TOF, becomes a feasible approach. Several techniques, including capillary electrophoresis [47,48,49,50], flow field-flow fractionation [51,52], and affinity separation via immunomagnetic beads [53,54] have been successfully applied for the identification of single bacterial species in poly-bacterial mixtures. As one example, Li and colleagues [55] found that the combination of a nonselective bacterial cell enrichment method (membrane filtration) followed by specific isolation of Gram-positive bacteria by vancomycin-conjugated magnetic particles resulted in the successful analysis of poly-bacterial mixtures from tap or reservoir water via direct MALDI-TOF methods. Similar approaches should be feasible for direct analysis of biological specimens (e.g., blood, CSF, or urine), thereby eliminating the need for time-consuming bacterial culture (see Figure 2, lower part).

Considerable effort has also been made toward developing methods for pathogen identification using High Performance Liquid Chromatography (HPLC)-MS. Similar to the separation techniques considered above, chromatographic separation of proteolytic digests of bacterial cell extracts via HPLC results in a significant reduction in background signals. The use of this method also provides an additional measure of confidence, as it facilitates sensitive detection and identification of unique peptide markers in a poly-bacterial mixture [56,57,58]. As one example, results from the study of Roux–Dalvai and colleagues [38] revealed that an HPLC-MS-based assessment of peptides, followed by the establishment of a set of unique peptide signatures using machine learning methods (i.e., top-down approach) resulted in the direct identification of fifteen distinct pathogenic bacterial species in urine specimens. Moreover, if the acquired peptide signatures are systematically evaluated using an in silico library generated from public databases (e.g., Swiss–Prot and Translated EMBL Nucleotide Sequence Data Library (TrEMBL); a bottom-up approach), the number of identifiable isolates can be increased to include more than 12,000 bacterial strains [59]. However, the transfer of these approaches to routine clinical laboratories remains limited and MALDI-TOF MS devices remain the most prominent at this time [38]. This finding holds true despite the successful application of HPLC-MS for the identification of single bacteria in a complex poly-bacterial mixture.

### 2.4. Biosensors

Pathogens can also be detected with highly specific biosensors [60,61]. Most biosensors are comprised of a recognition element, a transduction element, and a system for detection, amplification and quantification of output signals. The recognition element is required for specific detection of the pathogen and is designed to bind to the analyte of interest [62]. Biological recognition elements include antibodies, nucleic acids, receptors, enzymes, and cells or cell structures. Recognition elements are placed in direct contact with a suitable signal transducer. The transducer converts the biochemical binding event into a measurable signal; the most sensitive and accurate sensors rely on optical, electrochemical, or mass-based signal transduction [60,62,63]. Biosensors are designed to generate quantitative or semi-quantitative outcomes without the need for additional reagents, pre-enrichment or processing steps. Sensors must be housed in a self-contained and integrated system, which may then undergo further integration into a micro-fluidic system if required by the measurement approach used [64].

Biosensors based on mass transduction, including the quartz crystal micro-balance (QCM) and the surface acoustic wave (SAW), rely on the label-free detection of changes in mass. Here, piezoelectric crystals can serve as biosensors, as the analyzed frequency of oscillation depends on the electric frequency applied as well as on the mass of the crystal. The frequency of oscillation changes in response to the binding of analytes to the crystal or its functionalized surface (e.g., antibodies) [65]. QCM sensors have been used to detect *Mycobacterium tuberculosis*, including cells, bacterial antigens, and tuberculosis-associated cytokines [66]. The results of QCM-based analysis are comparable to those of gold standard techniques, such as Enzyme-linked immunosorbent assays (ELISA) and PCR, and the target selectivity is higher than those characterized for surface plasmon resonance (SPR) and potentiometric biosensors. However, complex matrices found in clinical samples frequently result in poor performance when these methods are used to detect analytes in whole blood, serum, and urine samples. Relatively high costs and problems with deliverability to end-users are also factors that limit the potential of QCM sensors for POC applications [66]. Other mass-based sensors, such as SAW have also been used to detect pathogens. Unfortunately, the use of this modality has also been associated with significant drawbacks, including relatively long incubation times, difficulties with crystal surface regeneration, problems with implementation into fluidic systems, and high packing costs [67].

Electrochemical sensors can detect the generation of electrochemically measurable processes or species (e.g., protons or hydrogen peroxide) and provide outputs based on changes in electrical properties resulting from interactions occurring at the sensor-sample matrix interface. Electrical parameters that can be analyzed with sensors include impedance, current (amperometric), voltage, and potential [62,64,68]. Amperometric-based sensors measure the current flow that reflects the concentration of a given analyte. To perform these measurements, a constant potential difference is applied between the electrodes, which results in a measurable flow of electrons that changes in the presence of an analyte [69]. Amperometric-based sensors have been developed that detect pathogens, including *Escherichia coli* [70,71,72]. Electrochemical impedance spectroscopy (EIS) is one of the most prominent examples of impedance-based biosensors. A small alternating voltage is applied across (surface-modified) electrodes over a wide range of frequencies and the resulting current between the electrodes passes through the sample. The specific binding of an analyte to modified electrodes results in a measurable change in the impedance [60]. Several different biosensors that detect differences in impedance have been developed, including several that are capable of identifying whole bacterial cells [73,74,75] and bacterial ribosomal RNA [76].

Optical biosensors have outstanding characteristics and are currently used widely. In addition to their high specificities and sensitivities, optical biosensors are small, cost-effective, and permit rapid real-time detection of numerous analytes [68,77]. The detection principles used in biosensor technology include reflection, refraction, absorption, infrared, Raman, chemiluminescence, fluorescence, or phosphorescence [65]. Depending on their detection mode, optical biosensor devices are classified as label-based (e.g., fluorophores and luminophores, enzymes, nanoparticles) or label-free. The introduction of a label is associated with some drawbacks, including the need for additional manipulation of the target analyte. As such, devices that use label-free techniques are preferable for most applications. Unfortunately, label-free detection systems are often not readily accessible [78]. Biosensors can be further categorized by their recognition mechanisms into affinity-based (i.e., specific binding of an analyte to a capture molecule) and catalytic-based sensors. In the latter case, chemical reactions are used to convert captured biochemicals into detectable products [77].

SPR-based biosensors include specific capture molecules that are immobilized on a glass plate and covered by a thin gold film which is irradiated from below by polarized light emitted from a hemispherical prism [79]. The resulting refractivity is measured as a function of the angle of incidence, which changes in response to specific analyte binding to the capture molecules. Antigen-antibody reaction kinetics can also be determined with SPR [68,80]. There are already numerous studies in which SPR has been used for the successful detection of bacteria, including *E. coli* [81], *Listeria monocytogenes* [82], *Salmonella* spp., *Staphylococcus aureus*, and *Vibrio cholerae* [80]. Moreover, SPR was used to detect products of bacterial metabolism, including neurotoxins from *Clostridium botulinum*, *E. coli* enterotoxin, and staphylococcal enterotoxins A and B [80]. However, these systems are large, complex, and expensive, and require specialized personnel; outcomes from SPR are also limited due to interference from non-specific binding [68]. As such, SPR may not be readily adapted into a POC device, although some progress has been made in this area due to the recent introduction of localized SPR and SPR imaging [83,84].

Whispering Gallery Mode (WGM) sensor technology is another important example of the use of an optical sensor for pathogen detection. Optical sensors based on WGM sensor technology have attracted much attention over the past decade. WGM is a promising concept that provides a basis for label-free detection of biomarkers of diverse substance classes, including proteins, nucleotides, and metabolites, as well as bacteria and viruses [85,86]. This technology utilizes a micro-resonator (e.g., micro-spheres, micro-rings, micro-disks) that confines light by Total Internal Reflection (TIR), thereby generating whispering gallery modes [87]. The refractive index of the environment of the micro-resonator determines the WGM positions, and the adsorption of molecules onto the surface of the micro-resonators results in a change in the effective refractive index. This interaction results in an extremely rapid and detectable shift in the mode position; this shift in the WGM spectrum can easily be monitored [78,88]. The surfaces of the micro-resonators can be functionalized with highly specific capture molecules for use in bioanalytical applications [89,90]. WGM sensors exhibit outstanding sensitivity and permit label-free detection at the level of single molecules or atoms in a very short timeframe [91,92].

As such, WGM sensors represent a promising technology that may be used to detect bacteria and bacterial infections directly from patient samples without the need for prior purification or amplification steps. Therefore, the time-to-result is determined largely by the biological processes involved in the specific binding of the target to the sensor. As such, entire tests can be performed within 30 min. WGM has been successfully used for the detection of viruses, including Influenza A [93,94], and MS2 [95]. his technique has also been used to detect the bacteria, *Helicobacter hepaticus* [78]. Detection of the bacterial species, *S. aureus*, was performed with a limit of detection of 5 pg/mL with micro-disks that carried staphylococcal-specific LysK endolysin as capture molecule. Importantly, the results of this trial were obtained within 15 min after sample application [96].

In addition to high sensitivity and short turnaround time, low manufacturing costs and the capacity to generate results from small sample volumes are additional favorable features of this technology that will facilitate its integration into clinically relevant settings [92]. Nonetheless, the transformation from the laboratory to a clinical diagnostic environment remains a major challenge and will require innovative solutions, especially with respect to sensor stability and specificity [91]. For these reasons, WGM sensors are not yet suitable for use in POC applications. However, there are several studies underway that involve a focus on improvements in the integration of photonic, plasmonic, micro-fluidic, and electronic components of these devices. WGM systems have also been successfully miniaturized into portable devices [85,97].

While both electrochemical and optical biosensors can be used to detect pathogens, each technology presents unique advantages and limitations. While optical techniques offer better sensitivity and specificity than electrochemical sensors, they have difficulties with processes that require analysis of turbid samples and with artifacts from quenching or interference from absorbing and fluorescing contaminants. Another factor that limits the utility of most optical sensor systems is the complex instrumentation required and the associated high costs of use. By contrast, electrochemical sensors are unaffected by the optical properties of target samples and can be used with relatively simple and low-power instrumentation. As such, the possibilities associated with the miniaturization of electrochemical sensors offer several major advantages for POC diagnosis. However, electrochemical biosensor systems are slightly more limited than optical biosensors with respect to sensitivity and specificity [60,68,98].

### 2.5. Technologies with Potential for POC Diagnosis of Bacterial Infections

**Figure 3:**
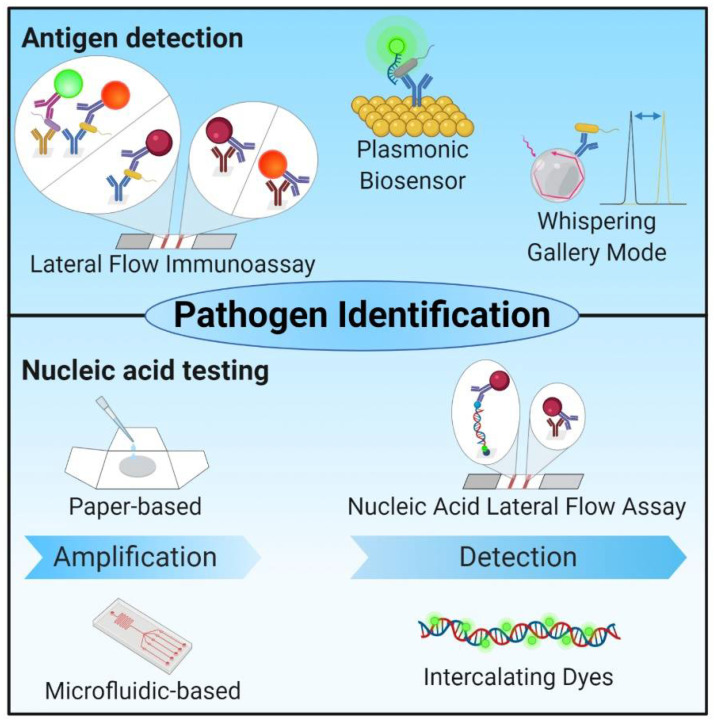
Illustration of different technologies for pathogen identification, suitable for point-of-care (POC) application. General approaches can be divided into antigen detection (**top**) and nucleic acid testing (**bottom**). Lateral Flow Immunoassays, for example, are readily applicable for detection of pathogen specific antigens, multiplexing-approaches for identification of several different species can be realized by incorporating different fluorescence labels (e.g., quantum dots). Furthermore, Plasmonic Biosensors show a great potential for POC applications, as sensitivity can be drastically increased and sample treatment can be avoided [105]. Another emerging technology for POC application is the Whispering Gallery Mode sensor technology, attracting much attention over the past decade. Here, the binding of molecules to the resonators surface can be detected as a change of the effective refractive index. Although the WGM technology displays a promising candidate, there are currently still several challenges hindering transformation into the clinical environment [78]. In case of approaches for POC applicable nucleic acid testing, several variable approaches are present. In general, shorter time periods during the amplification step can be achieved via implementation of paper-based (e.g., isothermal amplification [19]) or micro-fluidic- based (e.g., micro-fluidic PCR [106]) approaches. For detection of the amplicon, several different technologies can readily be used, including Nucleic Acid Lateral Flow Assays or intercalating dyes.

While the development of culture-independent molecular techniques has revolutionized the diagnosis of infectious disease (the currently most promising approaches are illustrated in Figure 3), there are several limitations associated with these methods. Most of the NAT methods require well-equipped clinical laboratories with sophisticated equipment, contain complex time-consuming operational protocols, and need critical technical expertise for effective processing and analysis of the results. The development of simple, rapid, easy-to-use, and inexpensive POC tests will beneficial toward providing clinical care in developed countries as well as in the resource-limited developing world [10,11]. Recent developments in lateral flow immunoassay (LFIA) techniques address all the criteria necessary for their application as POC diagnostic tests [99]. LFIA facilitates the identification of pathogens in a broad range of biological samples (e.g., whole blood, plasma, serum, saliva, and other sources) together with evaluation and confirmation of results by visual inspection [100]. LFIA technology relies on the binding of microbial antigens to specific capture antibodies immobilized on solid supports and utilizes colorimetric visualization provided by nanoparticle-antibody conjugates [101,102]. The development of multiplex biomarker detection within single LFIA platforms represents a significant milestone toward its use as a POC diagnostic, as this capacity facilitates rapid analysis of different stages of disease and diagnosis of multiple infections concurrently at reduced cost; the multiplex feature eliminates the need to perform each test separately [100]. However, several factors influence the signal resulting from a given sample (e.g., matrix effects and patient-to-patient variation) and a given environment (e.g., temperature and humidity). Multiplexing also presents the possibility (or probability) of antigen cross-reactivity; this limits the number and types of biomarkers that can be evaluated in a single assay. All of these factors can lead to relatively low accuracies and limited sensitivities described for LFIAs [100,102,103]. Another important drawback is that visual inspection does not allow for quantification. Furthermore, in cases of weakly-positive results, the outcome of the test may depend on the subjective interpretation of the operator, potentially leading to a high rate of false-negative or false-positive results [102,104].

Current research focuses on the use of different detection labels (e.g., magnetic particles, carbon nanoparticles, fluorescence micro-spheres, quantum dots, as well as up-converting phosphor or europium nanoparticles) and the employment of optical strip readers to detect and quantify read-outs, including magnetic, electrochemical, and chemiluminescence signals as well as fluorescence [101,107,108]. The introduction of optical strip readers has facilitated signal quantification; this development overcomes the drawbacks associated with an operator-dependent interpretation of test results and also improves the detectability and sensitivity of multiplex LFA sensors [42,102]. As mobile phones and smartphones become ubiquitous and part of everyday life around the world, the introduction of mobile LFIA readouts provides a tremendous opportunity with respect to their use as POC tests [109,110,111,112].

As an alternative to LFIAs, low-cost paper-based nucleic acid testing (NAT) devices have been developed that have substantially higher sensitivity and specificity than typically found in immunoassays. These tests include all three key steps required for accurate NAT, and combine the overall strong performance of conventional laboratory-based NATs (e.g., PCR) with the possibility for application at POC [19,113]. However, as of this writing, paper-based NAT technology has not completely fulfilled the ASSURED criteria recommended by the WHO and several challenges need to be met before bringing them to the marketplace [19,114]. Further improvements will be necessary, primarily directed at developing user-friendly operation steps, feasible storage conditions for all reagents, and the capacity for multiplex detection. Because many infectious diseases present with similar clinical symptoms, multiplex detection would facilitate differentiation between one or more causative agents [115,116]. The introduction of concepts and strategies based on emerging dynamic DNA nanotechnology, CRISPR Cas systems, and synthetic biology will promote the development of newer paper-based NATs with improved sensitivity, specificity, and accuracy for the diagnosis of a broader range of infectious diseases [117].

A particular approach, that would promote the use of NATs for POC applications, is the implementation of micro-fluidic techniques for nucleic acid amplification, thereby eliminating the need for sophisticated equipment (e.g., a thermocycler). Several amplification techniques have already been adapted to micro-fluidic formats, including RT-PCR [106,118,119,120,121], isothermal recombinase polymerase amplification (RPA) [122,123], NASBA [124,125] and LAMP [126,127]. Another advantage of micro-fluidic techniques is reduced time-to-result. For example, Nagatani and colleagues [106] identified an RT-PCR-based approach that reduced the amplification time to 15–20 min by using a continuous-flow chip, compared to the 1 to 2 h required by standard thermocyclers. PCR-based micro-fluidic systems use three different temperatures that correspond to the three amplification steps (i.e., denaturation, priming, and elongation); these can be achieved using a stationary chamber in combination with a variable heating device, or in different temperature zones together with combination with capillary flow [106,128]. Each approach has disadvantages. For example, RT-PCR performed using capillary flow can be complicated by the generation of air bubbles in the high-temperature zones. As such, other isothermal approaches (e.g., LAMP or RPA) may be more suitable for adaption to micro-fluidic formats. Nonetheless, as routine NAT-based diagnostic methods utilize RT-PCR, an adaptation of this technology to micro-fluidic systems may result in significantly more rapid availability of technology that can be used at POC. The establishment of a general and rapidly adaptable POC system using NAT strategies is of considerable interest, most notably for outbreak scenarios, such as observed during the German enterohemorrhagic *E*. *coli* (EHEC) epidemic in 2011; at this time, the most rapidly-developed methods for NAT are typically PCR-based. Once established, a POC system would not need to be limited to bacterial diagnostics but should be readily applicable for the diagnosis of fungal or viral diseases. In addition to ease in transferability, a strategy the ensures compatibility of the applied sample material is also of considerable interest. RPA is highly sensitive to the presence of detergents, including those used frequently in sample preparation [122]. As such, samples to be evaluated by RPA require more time-consuming preparation and purification techniques that ultimately hinder the adaption of this methodology for POC applications [16,125,126,127]. Taken together, RT-PCR approaches may be more favorable for adaptation to POC diagnostics, notably due to the robustness of PCR polymerases and the availability of single-step cell-lysis reagents [129].

In addition to NAT-based approaches, immunoassays should also be adaptable to micro-fluidic formats. The major factors complicating the transfer of this technology to POC application include the need for multistep reactions, limited sensitivity (in cases of colorimetric or fluorescent readout), and the need for a high level of sample purity when introducing a second signal amplification step (e.g., with reagents such as horseradish peroxidase (HRP)-linked immunoglobulins). In addition to classical antibody-based approaches, aptamers are evolving as useful tools for POC diagnosis and detection of corresponding immune responses [130]. A recent paper by Minopoli and colleagues [105] reported the use of a combination of aptamers with antibodies in a high-sensitivity plasmonic biosensor for the analysis of whole blood without any additional sample pretreatment. The introduction of a combination of recognition elements will widen the scope of POC applications for the diagnosis of infection and characterization of associated immune responses employing low-tech equipment such as mobile cameras [131]. However, sensitivity can be a major issue when using these applications to detect causal micro-organisms, as the immune response to even trace quantities of pathogenic bacteria can result in severe complications in sepsis [132].

**Table 1:** Comparison of methods applicable for pathogen detection. (RD: Resistance Determination, AST: Antimicrobial susceptibility testing, POC: Point-of-Care).

Method	Pathogen Identification (ID)	Time	RD	AST	Advantages and Disadvantages	POC	Ref
**Cell culture**	Growth based; all culturable bacteria	24–72 h cultivation + 18–24 h for biochemical ID	-	√	+ Cost-effective + Good specificity − Long turnaround times − Lacking sensitivity − Prone to errors in workflow − Difficulties with fastidious organisms − Unculturable organisms not detectable	-	[5,6,13,14,16,21,27,133,134,135]
**PCR-analysis and real-time PCR**	Sequence dependent amplification of bacterial genes > pathogen-specific	One to several hours	√	-	+ No cultivation + Good performance − Expensive − A priori knowledge on suspected pathogens necessary − Turnaround time − High-end instrumentation	-	[8,13,16,17,19,21,27,136,137,138,139,140,141,142]
**Next-generation sequencing**	Simultaneous sequencing of billions of nucleic acid fragments contained in heterogenous samples > identification on subspecies or strain level based on SNPs	14–20 h	√	-	+ Primer independent + Identification without a priori knowledge or suspicion + Faster adaption to new resistance mechanisms − Complex workflow with experimental pitfalls and biases − High overall error rate − Differentiation between colonization and infection critical	(√)	[5,22,23,24,27,28,29,31,32,33,136,143,144,145,146]
**MALDI-TOF; Direct sample testing**	Generated mass spectrum of molecular sample composition compared to spectral database containing spectra from pure colonies (pre-cultivation); Cell enrichment followed by specific isolation	2–50 h	(√)	(√)	+ Automatable + Low costs per test + Fast analysis − Pre-cultivation necessary − Several resistance mechanisms not detectable − Identification of subspecies limited − Polymicrobial analysis difficult + No pre-cultivation − A priori knowledge necessary	-	[15,38,39,41,43,44,45,46,47,48,49,50,51,52,53,54,55,147,148,149,150,151,152]
**HPLC-MS**	Separation of proteolytic digests of cell extracts via HPLC and identification of unique peptide markers	~4 h	-	-	− Transferability to routine lab remains limited	-	[38,56,57,58]
**Biosensors**	Recognition of pathogen presence or their metabolic activity via biological recognition elements in intimate contact to transducers and detection systems		√	-	+ (Semi-) quantitative measurement + No or few additional reagents, pre-enrichment or processing steps	(√)	[60,61,62,63]
**Mass transduction (e.g., QCM, SAW)**	Detection of mass changes on the sensor (e.g., piezoelectric crystals)	variable	(-)	(-)	+ Results comparable to ELISA, PCR + Target selectivity better than SPR − Affordability (QCM) − Long incubation times (SAW) − High packing costs (SAW)	-	
**Electrochemical transduction (e.g., EIS)**	Variable (e.g., screen-printed electrodes with antibiotic-seeded hydrogel or bacterial growth in electrode containing micro-wells in presence of antibiotics)	1–3 h	√	√	+ Unaffected of samples optical properties + Low-power instrumentation − Limited in sensitivity and specificity than optical-based sensors	(√)	[60,68,98,153,154]
**Optical transduction (e.g., SPR)**	Variable (e.g., digital time-lapse microscopy, SPR)	variable	√	√	+ High sensitivities and specificities + Sensors small and cost effective + Fast real-time detection − Label-based detection requires additional steps − Label-free detection often not easily accessible − Interference of non-specific binding − Trouble analyzing turbid samples − Interference in complex matrices	(√)	[60,68,77,78,98,155]
**Whispering gallery mode (optical)**	Label-free detection via capturing of pathogens and pathogen compounds with biological recognition molecules	~15–30 min	√	-	+ Label-free and real-time + Detection of single molecules and atoms + No prior purification or amplification + Low manufacturing costs + Small test volume − Sensor stability and specificity	(√)	[85,86,89,90,91,92,93]
**Lateral Flow Assays**	detection via capturing of pathogens and pathogen compounds with biological recognition molecules, detection with colorimetric and optical detection molecules	Several minutes	√	-	+ Broad range of biological samples + Results confirmed by naked eye − Low accuracy − Limited sensitivities − Cross-reactivity in multiplexing − Interpretation of weakly positive tests difficult	√	[99,100,102,103,104,109,110,111,112,156,157,158,159]
**Low-cost paper-based NAT**	Containing all three key steps of NAT for pathogen detection	45 min–120 min	√	-	+ Higher sensitivities and specificities than immunoassays + Capability for multiplex detection	(√)	[19,114,115,116]
**Micro-fluidic systems**	Variable (e.g., NAT-based micro-fluidic systems, chip-based isothermal nano calorimetry, micro-fluidic channels with gold-micro-electrodes, nanoliter-sized-micro-chamber and micro-array based micro-fluidic)	15 min–3 h	(√)	(√)	+ faster and better LOD by simple adaption to micro-fluidic format − sensitive to air bubbles − Sample preparation necessary (RPA)	(√)	[106,160,161,162,163]
**Biochemical tests (e.g., CarbaNP, BYG Carba test)**	No pathogen identification	Several minutes	√	-	− Applied amount of bacteria critical − Limitations in sensitivity for some lactamases	-	[164,165,166,167]

## 3. Resistance Profiling and Tests for Antimicrobial Susceptibility

The emergence of multidrug-resistant micro-organisms requires not only the rapid identification of causative pathogens but also the immediate determination of antimicrobial susceptibility and resistance [13]. In vitro antibiotic susceptibility testing (AST) is necessary for the selection of an optimally effective antibiotic regimen, and likewise to monitor and to prevent the spread of resistant organisms or resistance genes through the hospital and the community [17,57]. AST results facilitate the selection of suitable antibiotics and can identify a dosage that inhibits bacterial growth and decelerates the pace of emerging drug resistance [168].

An adequately-performing AST encompasses the many diverse modes of resistance, as micro-organisms feature a variety of biochemical mechanisms that can reduce susceptibility to previously-effective antibiotic regimens. Resistance can be acquired as a result of (i) mutations in genes that are associated with the mechanism of action of the antibiotic agent or by (ii) the acquisition of foreign DNA that encodes resistance determinants by horizontal gene transfer [169]. The biochemical mechanisms that promote antibiotic resistance include drug modification, drug target modification, active drug efflux (or decreased entry), and molecular bypassing [170]. Drug modifications are achieved when bacteria gain the capacity to express enzymes that destroy or modify the antimicrobial agent. Among the well-characterized examples of this phenomenon, bacteria can express β-lactam antibiotic-hydrolyzing β-lactamases or acetyltransferases, phosphotransferases, and nucleotidyltransferases that modify aminoglycoside antibiotics [171]. Similarly, point mutations in critical target bacterial genes can result in a change in amino acid sequence and alterations to protein structure; one or more of these changes might ultimately prevent antibiotic binding and thus its anti-bacterial effects. Changes in drug targets can also be induced by enzymes that catalyze highly efficient and regionally-selective modifications [170]. For example, most antimicrobial agents must cross the bacterial cell wall to reach their site of action; metabolic changes can serve to restrict outer membrane permeability, thereby preventing the intracellular accumulation of drugs in sufficient concentrations. Restricted permeability can result from the loss of porin proteins that facilitate antibiotic transfer across the outer membrane; likewise, overexpression of outer membrane proteins can prevent antibiotic binding [172]. Another strategy that promotes antibiotic resistance involves the over-expression of efflux pumps that remove antibiotics from the intracellular milieu [173]. Likewise, overproduction of the drug target may allow the bacteria to “bypass” the metabolic pathway originally subject to antibiotic-mediated inhibition. Bacteria can also compensate for antibiotic-mediated inhibition at the primary target by forming new targets that perform similar biochemical functions [169]. As such, technologies used for resistance determination and ASTs must be versatile to facilitate identification of a wide variety of antibiotic resistance mechanisms.

### 3.1. Culture-Based Methods

The current technologies used for in vitro resistance testing and AST include culture, molecular, and spectrometry-based approaches; culture-based methods remain the gold standard [27,154]. Traditional ASTs rely on microbial cultivation methods, including 96-well micro-broth dilution, agar dilution, and disk diffusion strategies. Valuable results can be obtained using these methods to screen for antimicrobial susceptibility, and all except for the disk diffusion method provide a quantitative evaluation of minimum inhibitory concentrations (MICs) [27,133]. Using these methods, susceptibility or resistance is determined by visual examination of bacterial growth in the presence of antimicrobial agents at various concentrations [133]. However, these strategies do not permit a more precise characterization of existing resistance mechanisms. Moreover, these growth-based tests are limited by turnaround times that range from 12 to 72 h; variability in inoculum size or culturing conditions can result in poor accuracy [21,134,135]. This method is also not suitable for the evaluation of slowly-growing or uncultivable pathogens [174].

### 3.2. Molecular Detection, Genetic Methods, RNA Markers, and Sequencing-Based Methods

Molecular-based approaches are primarily focused on amplification or hybridization of (over)expressed microbial genetic sequences that encode specific resistance determinants (e.g., genes that encode drug-modifying enzymes), using conventional polymerase chain reaction (PCR), quantitative RT-PCR, or DNA-micro-arrays. These methods provide great precision, sensitivity, and specificity with comparatively shorter turnaround times of ~1–6 h [13,27,140]. Multiplex PCR and digital PCR are examples of emerging technologies in this field [141]. Several multiplex PCR assays have been developed that include panels for detection of different resistance genes; use of these assays substantially reduces the time to clinically actionable results [175] Currently available multiplex PCR panels include Biofire®-FilmArray® (biomérieux) and Acuitas® AMR gene panel and Unyvreo System (OpGen Inc., Gaithersburg, MD, USA/Curetis GmbH, Holzgerlingen, Germany) [142]. As such, mechanisms underlying antimicrobial resistance can be detected via the amplification of resistance genes well before the results of traditional culture-based methods would be available [18]. A large number of cells obtained by culture enrichment are required to obtain the amount of DNA needed to achieve robust PCR performance, especially when targeting low-abundance genes and mechanisms as well as heteroresistance [26,145,146,147]. Although several culture-independent methods have already been developed to address these issues, these methods feature problems that include limited and variable clinical sensitivities [136]. It is possible to detect low-abundance targets and heteroresistance using digital PCR systems, which permit immediate analysis of samples without the need for prior culture enrichment [146,147,148,149].

However, there are caveats and pitfalls when screening is limited to certain genes and resistance determinants. Among these concerns, false-positive tests can result from the amplification of silent genes or pseudogenes. Similarly, mutations in primer binding sites may generate false-negative results by hampering PCR amplification. Finally, current genetic methods cannot address the possibility of new and as-yet-uncharacterized resistance mechanisms, as the PCR assays focus on the detection of previously-identified genes and genetic determinants. It is difficult to detect highly variable organisms and complex, rapidly evolving mechanisms with conventional PCR, especially Gram-negative species that include extended-spectrum β-lactamase (ESBL) producing and carbapenem-resistant Enterobacteriaceae (CREs) which can differ from one another by single-nucleotide polymorphisms (SNPs) [141,142]. In these strains, PCR-detection of resistance markers and phenotypic resistance are not always correlated with one another [139].

In recent years, the increased availability of more affordably-priced sequencing technologies, including whole-genome and NGS, has provided a means to evaluate an entire bacterial genomic DNA sequence; this strategy facilitates confirmation of a bacterial species and identification of potential resistance genes at comparatively low cost [136,143,144]. The sequence of the entire genome of an organism provides a profound understanding of its functional potential; as such, this is an attractive approach for antimicrobial resistance testing. Public databases already contain a considerable amount of data that can be used to facilitate screening for antimicrobial resistance determinants in newly-acquired sequencing data [174]. This is a primer-independent technology that provides more rapid identification of new resistance mechanisms than can be achieved using DNA amplification or hybridization-based assays targeting pre-identified genes [143,144]. However, sequencing technologies are also limited, as they can only serve to reveal previously-identified resistance mechanisms. Nonetheless, as new resistance mechanisms are discovered and added to the database, subsequent sequencing results can be checked immediately against these findings [144,176]. Drawbacks associated with this technology include complex workflows with experimental pitfalls and biases inherent in current NGS protocols, as well as slow turnaround times [22,27,28,136]. The MinION nanopore-based sequencing method is an emerging technology that could facilitate rapid sequencing for antibiotic resistance determinants. The potential of the MinION sequencer was demonstrated in a study focused on the identification of Gram-negative bacteria and the confirmation of an ESBL phenotype, and likewise on the elucidation of the complete genome sequences and antimicrobial susceptibility of *Neisseria gonorrhoeae* [177,178]. Although NGS technologies facilitate the identification of characterized antibiotic resistance genes and mechanisms, it is not usually possible to draw conclusions regarding antimicrobial susceptibility from sequence data alone. Attempts are being made to generate algorithms to predict antimicrobial susceptibility [151,152,153] and to establish machine learning-based antibiotic susceptibility profiles from whole-genome sequencing data [145,146]. However, these directions are still at an early phase, and unavailable for clinical use.

### 3.3. MALDI-TOF MS-Based

MS has become a method of choice for mechanistic elucidation and characterization of small-molecule—protein interactions, including interactions with β-lactam antibiotics or with the commonly used β-lactamase inhibitor, clavulanic acid [147]. As such, it is reasonable to consider extending MS-based techniques for the profiling of antimicrobial resistance. As MALDI-TOF MS is used frequently for the identification of bacterial species, and the necessary devices and equipment are often present in routine clinical laboratories, considerable effort has been made toward the use of this modality for the detection of antimicrobial resistance patterns. At current writing, several different approaches have been described that address this issue, including the identification of a “resistance peak pattern,” detection of β-lactam hydrolysis products, quantification of incorporated stable isotope-labeled amino acids, and quantification of bacterial growth in presence of one or more antibiotics [148]. The latter three approaches were tested exclusively on the MALDI Biotyper CA System (Bruker Daltonics, Inc.), which restricts their overall applicability given the limited availability of this specific device [160,161,162,163]. Nonetheless, commercial solutions (in vitro device (IVD)-CE certified) have been developed for this application (e.g., MBT-STAR Assays), which have facilitated the detection of active carbapenemases, cephalosporinases, and β-lactamases [148]. Unfortunately, commercial solutions that were developed for assays involving stable isotope-labeled amino acids (MBT-RESIST Assay) or quantification of bacterial growth (MBT-ASTRA) are no longer available on the vendor website [148]. Despite the restrictions associated with the device, MALDI-TOF-based antimicrobial resistance profiling is superior to nucleic acid-based approaches, as it can be used to detect the presence of the active enzyme, as opposed to the presence of its corresponding genes [149]. However, given the overall complexity and heterogeneity of bacterial resistance mechanisms and the need for cultivation of the biological specimen, the clinical utility of these approaches remains limited.

### 3.4. Innovative and Rapid Testing Systems: Efforts toward POC Testing of Antimicrobial Resistance

**Figure 4:**
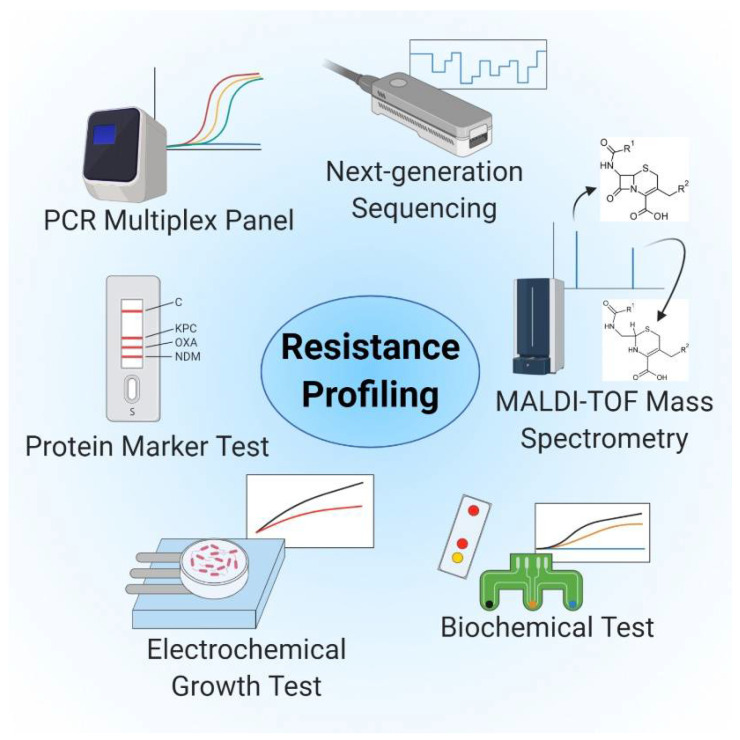
Overview of current and promising technologies suitable for antimicrobial resistance profiling. Beside classical culture-based approaches (not included), currently available commercial solutions include PCR multiplex panels (e.g., Biofire^®^-FilmArray® panels (biomérieux)), MALDI-TOF MS (MTB-STAR Assays (Bruker Daltonics, Inc.)), biochemical tests (e.g., RAPIDEC® CARBA NP test (biomérieux)) and protein marker tests (e.g., RESIST-3 O.K.N. *K*-SeT (Coris BioConcept, Gembloux, Belgium)). Further promising technological advances have been made in the field of Next-generation Sequencing (e.g., [174]) adaption of biochemical tests to faster electrochemical formats (e.g., [167]) or development of electrochemical sensors for bacterial growth (e.g., [154]). Several of these approaches are suspected to result in commercially available solutions for antimicrobial resistance profiling in the future.

Antibiotic therapy is initially empiric, as the results of antimicrobial resistance and susceptibility testing are usually not available at the time that therapy is initiated. Ideally, one would prefer to have susceptibility profiles available as early as possible, especially when asked to provide care for critically ill patients [154,179]. POC antimicrobial resistance tests will need to be inexpensive and largely automated; they will need to accommodate low volumes of material to be tested and to provide MIC values in a multiplex mode for a large cohort of available antibiotics (the currently available and most promising approaches are illustrated in Figure 4). The methods discussed earlier all meet some of these specifications, but none achieve success when considering all of these criteria [141].

Biosensors utilize small test volumes and may provide insight into distinct resistance mechanisms. As such, this methodology may combine both rapid and sensitive detection of resistance-associated genes and offer the opportunity to be integrated into or used alone as a POC test. Huang and colleagues [180] reported that electrochemical technologies, such as EIS, can promote direct, amplification- and label-free detection of *bla_NDM_* plasmid genes, including the New Delhi metallo-β-lactamase via hybridization. Other electrochemical-based tests have been developed to facilitate the detection of the resistance gene, *mecA* [181,182,183]. Although these tests provide rapid detection of critical resistance-associated genes, they do not specify antibiotic susceptibility.

Antimicrobial resistance and susceptibility testing can also be performed by determining protein-, enzyme-, antigen-, and metabolite-based molecular signatures and processes using spectrometry techniques, biosensors, and immunoassays. The use of these methods confirms that a detected resistance gene is also expressed and phenotypically present. 

Biochemical tests can be applied to detect resistance-associated enzymes such as carbapenemases. The Rapidec Carba NP test is based on detection of in vitro hydrolysis of the β-lactam ring of imipenem by carbapenemases, which results in a color change on a pH-indicator [184,185]. Further development of this test (Carba NP test II) has facilitated discrimination between different classes of these enzymes [186]. However, the results are directly dependent on the number of bacterial cells provided for evaluation; transfer of culture from a plate using an inoculation loop is required to carry out the test [187]. Likewise, the assay has limited sensitivity for certain lactamases, notably OXA-48 and some metallo-β-lactamases [170,171,172]. The BYG Carba test also analyzes changes in pH and redox activity that result from the enzymatic hydrolysis of imipenem. In this assay, modifications in local conductivity are monitored using an electrochemical biosensor integrated into a portable potentiostat. This assay provides a shorter time-to-result than the colorimetric-based method and the test can be performed at room temperature. However, this method also requires a cultivation step and is initiated with an inoculation loop of bacterial culture [167] and provides no assessment of antibiotic susceptibility.

Several LFIAs have been developed that are capable of direct detection of protein markers of resistance, including the various types of β-lactamases. These tests typically involve specific capture antibodies and detection antibodies conjugated with colloidal gold, and outcomes are evaluated by visual inspection [174,175,188,189]. However, detection of different classes of β-lactamases often requires more than one test strip. Similarly, the results provide no information on the therapeutic efficacy of different antibiotics. In order to determine various types of resistance and resistance mechanisms using a single test, a combination of LFIAs and micro-arrays might be employed. Identification and biochemical analysis of pathogens and antibiotic resistance could be conducted via a large number of spatially separated test zones. A single LFA test strip could thus be used for rapid detection of the most widespread and critical resistant bacteria (e.g., methicillin-resistant *S. aureus* (MRSA), vancomycin-resistant enterococci (VRE), ESBL-producing Enterobacteriaceae and CRE) together with data on antibiotic resistance patterns.

Micro-fluidic systems may also be used for POC tests for specific applications. Micro-fluidic devices have been combined with several different technologies to detect antimicrobial resistance and antimicrobial susceptibility. For example, magnetic nanoparticles linked to antibodies specific for the resistance factor, penicillin-binding protein 2a, were used to detect MRSA that were captured in a micro-fluidic device. Detection was achieved by electrochemical detection using strain-specific antibodies linked to alkaline phosphatase. The limit of detection using this method was 845 colony-forming units (CFU)/mL in patient-derived nasal swabs; results indicated that this strategy facilitated discrimination between pathogen and common nontarget nasal flora within a turnaround time of under 4.5 h [161].

While detection of resistance determinants remains important, it is also critical to identify all antibiotics that remain effective. As such, rapid technologies have been developed that address several strategies that might be used for AST. One rapid AST for *Bacillus anthracis* uses an optical method and automated digital time-lapse microscopy to evaluate the growth and morphological impact of relevant antibiotics [155]. As this test can be performed within 4 h and is thus 75% faster than conventional methods, it is still not suitable for the use as a POC test. As is the case with several other novel methods, microscopic monitoring of bacterial growth may require complex optical screening instruments [190].

Several electrochemical-based test systems have emerged as a more practical and potentially cost-effective alternative as a POC application. A low-cost diagnostic sensor test that includes a screen-printed gold-electrode modified with an antibiotic-seeded hydrogel was used for electrochemical monitoring of the growth of *E. coli* in the presence and absence of streptomycin over ~2.5 h [154]. Another electrochemical method that facilitated rapid phenotypic profiling of antibiotic-resistant bacteria within a turnaround time of 1 h has been reported. In this approach, bacteria are captured in miniaturized electrode-containing wells and incubated with antimicrobial agents. Bacteria that remain metabolically active can be identified by electrochemically monitoring the reduction in a redox-active reporter molecule [153].

The time required to generate AST results can be significantly reduced by adaption to micro-fluidic systems; as the bacteria are confined to a smaller volume, cell division can be detected earlier and at lower limits of detection. The potential of micro-fluidic systems for rapid AST has been illustrated by several studies [168,190,191]. The potential for rapid AST was evaluated with an integrated chip-based isothermal nano calorimetry platform. In this setting, metabolic activity and antimicrobial action of antibiotics provided at subinhibitory concentrations were detected in real-time by monitoring the heat generated by the bacterial cells. The proof-of-concept study provided MIC values for 3 clinically relevant antibiotics within a few hours using *E. coli* as model organism [161]. Furthermore, a combination of micro-fluidic channels with gold-micro-electrodes was used in an electrochemical micro-fluidic chip to facilitate automation of antibiotic mixing and its distribution into multiple test chambers. This test provided a rapid bacterial count together with AST of *E. coli* in 170 and 150 min, respectively [162]. In another study, the use of a nanoliter-sized micro-chamber and micro-array-based micro-fluidic system resulted in rapid AST and MIC determinations for several different antibiotics. Nanoliter-sized chambers were loaded with bacterial suspensions, both with and without antibiotics, and the fluorescence emitted from the reduction in resazurin (correlating with bacterial growth) was measured. The method had been tested with numerous wild-type clinical bacterial isolates including *E. coli, Klebsiella pneumoniae*, and *Enterococcus faecalis*. Test results were available between ~1–3 h, depending on the growth rate of the bacterial species [163]. With further miniaturization of micro-fluidic systems and, for example, the replacement of active with passive pumps, micro-fluidic systems become easier to operate and more suited for use as POC devices.

Although there have been many efforts directed toward the development of POC-AST devices, most of these methods typically meet some of the aforementioned specifications; however, none of these devices is as yet fully satisfactory. A combination of different technologies together with the addition of new ideas and strategies will be needed to generate clinically applicable POC tests.

## 4. Conclusions and Outlook

In recent years, considerable progress has been made in developing technologies for the detection of infectious diseases. Culture-independent methods, including NATs and NGS-based strategies, have revolutionized the field of infectious disease diagnostics by providing rapid results and facilitating the detection of uncultivable pathogens. Despite these extraordinary technological advances, challenges remain to be addressed. Approved molecular tests are currently available for only a limited number of pathogens; furthermore, these diagnostics are notably underutilized [3]. Major limitations of these tests include slow turnaround time, poor test performance, limited access to testing materials, and high cost. The ideal diagnostic test would be inexpensive, accurate, easy to perform using tools and resources that were available to all, would provide results rapidly without a priori knowledge of likely causative agents, and would guide appropriate options for antimicrobial therapy. POC tests, including LFIAs, biosensors, and paper-based NAT devices, reveal a significant potential to fulfill all these requirements. However, at this writing, available tests based on these detection strategies are far from ideal.

In the coming years, antibiotic-resistant pathogens will emerge in increasing incidence and complexity, thereby posing an enlarging challenge for healthcare systems worldwide [192]. At this time, culture-based methods remain important modalities used for both phenotypic characterization and antimicrobial susceptibility testing. However, more rapid methods will be needed, particularly those directed at the identification of microbial antibiotic resistance, as one will need to have immediate results in order to provide adequate isolation and treatment strategies for infected patients. More rapid tests would optimize patient outcomes by decreasing the use of partially effective and ineffective antimicrobial agents and reduce the risk of developing drug-resistant organisms.

In summary, there is still a considerable need for new options for diagnostics and diagnostic strategies to be used for the detection of pathogens and characterization of bacterial infections. Emerging techniques, including host-based diagnostics [13,193,194], synthetic biology (e.g., phage-based diagnostics [195], CRISPR and Cas systems [196,197]) and those relying on AI and machine learning [37,198] all have the potential to advance our understanding and capabilities when used to develop new infectious disease diagnostics.

## Data Availability

No new data were created or analyzed in this study. Data sharing is not applicable to this article.

## Abbreviations

POC	Point-of-Care
ASSURED	Affordable, Sensitive, Specific, User-friendly, Rapid and Robust, Equipment-free, and Deliverable to end-users
WHO	World Health Organization
PCR	Polymerase Chain Reaction
RT-PCR	Reverse Transcriptase Polymerase Chain Reaction
LAMP	Loop-mediated isothermal amplification
NASBA	Nucleic acid sequence-based amplification
TMA	Transcription-mediated amplification
NAT	Nucleic acid testing
NGS	Next-generation sequencing
mNGS	Metagenomic Next-generation sequencing
SNPs	Single nucleotide polymorphisms
CSF	Cerebrospinal fluid
IBMA	Isolate-based mixture assessment
HPLC	High-Performance Liquid Chromatography
QCM	Quartz crystal microbalance
SAW	Surface acoustic wave
ELISA	Enzyme-linked immunosorbent assay
SPR	Surface plasmon resonance
EIS	Electrochemical impedance spectroscopy
WGM	Whispering Gallery Mode
TIR	Total Internal Reflection
LFIA	Lateral Flow Immunoassay
RPA	Isothermal recombinase polymerase amplification
EHEC	Enterohemorrhagic *E. coli*
HRP	Horseradish peroxidase
AST	Antibiotic susceptibility testing
MIC	Minimum inhibitory concentration
ESBL	Extended-spectrum β-lactamase
CRE	Carbapenem-resistant Enterobacteriaceae
VRE	Vancomycin-resistant enterococci
